# From gatekeepers to regenerators: the plasticity of Paneth cells

**DOI:** 10.1038/s44318-026-00906-w

**Published:** 2026-08-30

**Authors:** Somara De Beul, Claude Libert, Tineke Vanderhaeghen

**Affiliations:** 1 VIB Center for Inflammation Research, VIB, Ghent, Belgium; 2 Department of Biomedical Molecular Biology, Ghent University, Ghent, Belgium

**Keywords:** Microbiology, Virology & Host Pathogen Interaction, Signal Transduction, Stem Cells & Regenerative Medicine

## Abstract

A recent study identifies CDC42 as a regulator of ROS-balance-dependent Paneth cell identity and antimicrobial function.

**Figure:**
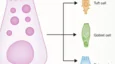

## Teaser

Paneth cells (PCs) are secretory cells in the small intestine with functions in intestinal homeostasis and innate immunity, with recent studies showing their ability to dedifferentiate and contribute to epithelial regeneration after injury. In this issue of *The EMBO Journal*, Yang et al, (2026) identify reactive oxygen species (ROS) as a central determinant of PC identity and uncover the Rho-family GTPase CDC42 as a critical regulator of this process.

Paneth cells (PCs) occupy a unique position in the small intestinal epithelium, where they reside at the base of the crypts of Lieberkühn, adjacent to intestinal stem cells (ISCs). PCs are known for their dual role: they support the neighboring ISCs by secreting niche factors, while simultaneously protecting the crypts from microbial invasion by releasing antimicrobial peptides (AMPs), including α-defensins and lysozyme. These activities are essential for maintaining intestinal homeostasis, epithelial integrity, and limiting pathogen colonization. Consequently, PC identity loss is reported in a variety of intestinal diseases, including Crohn’s disease, graft-versus-host disease, necrotizing enterocolitis, and enteric infections with *Salmonella Typhimurium* (*S. Typhimurium*), where it is associated with disease severity and poor clinical outcome (Quintero and Samuelson, 2025). Although inflammatory mediators such as interferon-γ (IFN-γ) induce PC degranulation and extrusion, how inflammation causes PC identity loss remains incompletely understood (Wallaeys et al, 2024). For decades, PCs were viewed as terminally differentiated secretory cells. This view has changed due to the fact that PCs retain remarkable plasticity. Following injury or depletion of the ISC compartment (Schmitt et al, 2018; Yu et al, 2018), they can dedifferentiate, reacquire progenitor-like properties, and contribute to epithelial regeneration (Quintero and Samuelson, 2025). These observations raise an important question: what signals trigger the transition from a mature PC with antimicrobial activities towards regenerative PC progenitors under pathological conditions?

In this issue of *The EMBO Journal*, Yang and colleagues (2026) identify reactive oxygen species (ROS) as a suppressor of PC differentiation, and reveal the Rho-family GTPase CDC42 as the factor counteracting ROS to promote PC identity. They have shown that oxidative stress does not only induces PC dysfunction during inflammation, but it also directly stimulates mature PCs to proliferate and dedifferentiate. Furthermore, they propose that CDC42 maintains PC antimicrobial function by preventing excessive ROS accumulation. In contrast, elevated ROS levels promote PC dedifferentiation, reducing AMP production while enabling cell cycle re-entry and multipotency. These findings reveal a trade-off between the specialized antimicrobial activity of PCs and their cellular plasticity. Single-cell transcriptomics analysis shows that PCs exist along a maturation trajectory. Immature PCs display high oxidative phosphorylation (OXPHOS) and ROS-associated transcriptional programs. As PCs mature, they progressively lose this OXPHOS signature while gaining their antimicrobial activity characterized by high AMP production (Yang et al, 2026). These observations highlight the importance of mitochondrial metabolism in shaping PC identity and build further on earlier work by Rodríguez-Colman and colleagues (2017), who showed that metabolic interactions between PCs and neighboring ISCs regulate crypt homeostasis (Rodr**í**guez-Colman et al, 2017). While ROS have previously been linked to ISC regulation, Yang and colleagues (2026) position oxidative stress as a determinant of PC identity, thereby linking mitochondrial metabolism to the balance between its antimicrobial activity and PC plasticity.

A key question was whether ROS only mark immature PCs or actively contribute to cell fate decisions. Using pharmacological approaches to increase ROS levels, Yang et al (2026) show that oxidative stress drives mature PCs to re-enter the cell cycle and generates PC progenitors that differentiate into enterocytes, goblet cells, and tuft cells. Importantly, antioxidant treatment abolishes these effects, providing a causal role for ROS in driving PC plasticity. Although IFN-γ similarly stimulates PC proliferation, it fails to generate comparable lineage-tracing patterns, indicating that not all forms of PC proliferation are equivalent and that ROS activate a distinct regenerative program (Yang et al, 2026). These findings identify oxidative stress as a molecular switch that enables mature PCs to acquire stem-like properties.

How oxidative stress initiates this regenerative program in PCs remains an important open question. One potential explanation involves the activation of YAP and TAZ, the transcriptional effectors of the Hippo pathway, which are recognized as central regulators of epithelial regeneration (Meyer et al, 2022). Following tissue injury, YAP/TAZ signaling promotes transcriptional programs that enable differentiated epithelial cells to reacquire a regeneration potential. Interestingly, CDC42 has also been shown to regulate epithelial polarity and proliferation through a YAP-EGF-mTOR signaling axis (Zhang et al, 2022), raising the possibility that oxidative stress, CDC42 signaling, and YAP activation might orchestrate PC reprogramming. Although this possibility remains speculative, it offers a base for future mechanistic studies. Yang and colleagues (2026) further identify CDC42 as a key regulator of PC identity. Loss of CDC42 increases intracellular ROS, suppresses AMP production, disrupts PC localization, and promotes cell cycle re-entry and dedifferentiation. Importantly, many of these defects are recapitulated in PC-specific CDC42 knockout mice, demonstrating that CDC42 mainly acts in a cell-autonomous manner to preserve PC identity (Yang et al, 2026). By linking CDC42 to oxidative stress, this study provides a mechanistic connection between cellular metabolism and regenerative reprogramming.

**Figure 1:**
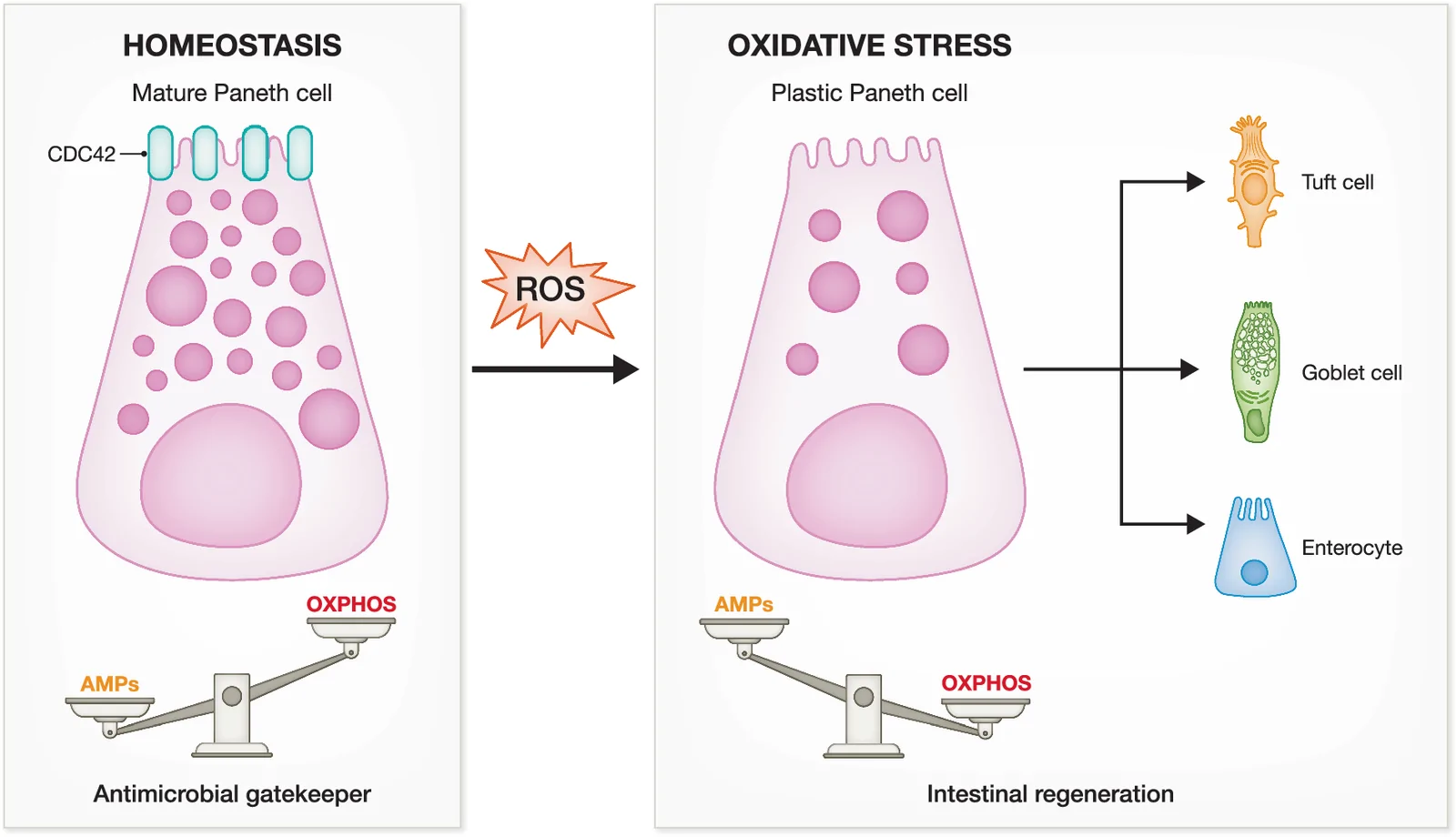
From antimicrobial gatekeepers to regenerators. Upon homeostasis, mature Paneth cells are known for their antimicrobial activity that coincides with a decline in their oxidative phosphorylation (OXPHOS) capacity. Upon exposure to reactive oxygen species (ROS), the antimicrobial activity of Paneth cells declines and elevates the plasticity of Paneth cells. This results in the proliferation and dedifferentiation of Paneth cells into tuft cells, goblet cells, and enterocytes. One of the key regulators of this balance is the Rho GTPase CDC42, loss of which dramatically increases ROS levels.

The physiological relevance of this pathway becomes clear during enteric infection. PCs are a critical component of the intestinal antimicrobial barrier, and *S. Typhimurium* is known to suppress AMP expression, suggesting that disruption of PC function may represent an important strategy to evade host defense mechanisms (Salzman et al, 2003). Consistent with this idea, Yang and colleagues (2026) show that disrupting the CDC42-ROS axis compromises this defense: mice lacking CDC42 exhibit reduced AMP production and are more susceptible to *S. Typhimurium* infection, whereas constitutive activation of CDC42 preserves PC identity and confers greater resistance to infection (Yang et al, 2026). These findings demonstrate that maintaining PC identity has direct consequences for host defense. Conceptually, the work reframes how PC dysfunction is interpreted during intestinal inflammation. Loss of mature PCs has been viewed as a passive consequence of epithelial injury. The findings of Yang and colleagues (2026) instead propose that it represents an adaptive trade-off. Here, oxidative stress functions as a molecular switch that shifts PCs away from their antimicrobial activities towards tissue regeneration, allowing damaged crypts to replenish the epithelial barrier. Rather than existing as terminally differentiated cells with fixed functions, PCs emerge as dynamic cells capable of balancing two competing biological priorities: antimicrobial defense and tissue regeneration (Fig. 1).

Together, this study identifies the CDC42-ROS axis as a key regulator of PC fate, linking redox homeostasis to the balance between antimicrobial defense and epithelial regeneration. The study is strengthened by complementary loss- and gain-of-function models that connect CDC42 activity, oxidative stress, and susceptibility to enteric infection. Nevertheless, some limitations should be considered. Since *Lyz1*-driven Cre activity is not entirely restricted to PCs, contributions from other LYZ1-expressing cell types cannot be fully excluded. In addition, the proposed OXPHOS-ROS model is established in mice and would benefit from validation in human tissues. Future studies should determine how oxidative stress activates regenerative signaling pathways, whether YAP/TAZ or other stress-responsive pathways are involved, and whether the modulation of this CDC42-ROS axis can be therapeutically used in intestinal diseases. By positioning oxidative stress as a determinant of PC fate rather than a consequence of inflammation, Yang and colleagues (2026) provide a new framework for understanding how the intestinal epithelium balances tissue repair with antimicrobial defense.
